# Characterization of Endogenous SERINC5 Protein as Anti-HIV-1 Factor

**DOI:** 10.1128/JVI.01221-19

**Published:** 2019-11-26

**Authors:** Vânia Passos, Thomas Zillinger, Nicoletta Casartelli, Amelie S. Wachs, Shuting Xu, Angelina Malassa, Katja Steppich, Hildegard Schilling, Sergej Franz, Daniel Todt, Eike Steinmann, Kathrin Sutter, Ulf Dittmer, Jens Bohne, Olivier Schwartz, Winfried Barchet, Christine Goffinet

**Affiliations:** a Institute of Experimental Virology, TWINCORE Centre for Experimental and Clinical Infection Research, Hannover, Germany; b Instituto de Ciências Biomédicas Abel Salazar, Universidade Do Porto, Porto, Portugal; c Institute of Clinical Chemistry and Clinical Pharmacology, University Hospital, University of Bonn, Bonn, Germany; d Institut Pasteur, Department of Virology, Virus and Immunity Unit, Paris, France; e Institute of Virology, Hannover Medical School, Hannover, Germany; f Department of Molecular and Medical Virology, Ruhr University Bochum, Bochum, Germany; g Institute for Virology, University Hospital Essen, University of Duisburg-Essen, Essen, Germany; h German Center for Infection Research (DZIF), Cologne-Bonn, Germany; i Institute of Virology, Campus Charité Mitte, Charité-Universitätsmedizin Berlin, Berlin, Germany; j Berlin Institute of Health, Berlin, Germany; Emory University

**Keywords:** HIV-1, interferons, antiviral factor, SERINC5, Nef, CRISPR/Cas9, human immunodeficiency virus

## Abstract

When expressed in virus-producing cells, the cellular multipass transmembrane protein SERINC5 reduces the infectivity of HIV-1 particles and is counteracted by HIV-1 Nef. Due to the unavailability of an antibody of sufficient specificity and sensitivity, investigation of SERINC5 protein expression and subcellular localization has been limited to heterologously expressed SERINC5. We generated, via CRISPR/Cas9-assisted gene editing, Jurkat T-cell clones expressing endogenous SERINC5 bearing an extracellularly exposed hemagglutinin (HA) epitope [Jurkat *SERINC5*(iHA knock-in) T cells]. This modification enabled quantification of endogenous SERINC5 protein levels and demonstrated a predominant localization in lipid rafts. Interferon alpha (IFN-α) treatment enhanced cell surface levels of SERINC5 in a ruxolitinib-sensitive manner in the absence of modulation of mRNA and protein quantities. Parental and *SERINC5*(iHA knock-in) T cells shared the ability to produce infectious wild-type HIV-1 but not an HIV-1 Δ*nef* mutant. SERINC5-imposed reduction of infectivity involved a modest reduction of virus fusogenicity. An association of endogenous SERINC5 protein with HIV-1 Δ*nef* virions was consistently detectable as a 35-kDa species, as opposed to heterologous SERINC5, which presented as a 51-kDa species. Nef-mediated functional counteraction did not correlate with virion exclusion of SERINC5, arguing for the existence of additional counteractive mechanisms of Nef that act on virus-associated SERINC5. In HIV-1-infected cells, Nef triggered the internalization of SERINC5 in the absence of detectable changes of steady-state protein levels. These findings establish new properties of endogenous SERINC5 expression and subcellular localization, challenge existing concepts of HIV-1 Nef-mediated antagonism of SERINC5, and uncover an unprecedented role of IFN-α in modulating SERINC5 through accumulation at the cell surface.

**IMPORTANCE** SERINC5 is the long-searched-for antiviral factor that is counteracted by the HIV-1 accessory gene product Nef. Here, we engineered, via CRISPR/Cas9 technology, T-cell lines that express endogenous *SERINC5* alleles tagged with a knocked-in HA epitope. This genetic modification enabled us to study basic properties of endogenous SERINC5 and to verify proposed mechanisms of HIV-1 Nef-mediated counteraction of SERINC5. Using this unique resource, we identified the susceptibility of endogenous SERINC5 protein to posttranslational modulation by type I IFNs and suggest uncoupling of Nef-mediated functional antagonism from SERINC5 exclusion from virions.

## Abstract (precis)

SERINC5 is the long-searched-for antiviral factor that is counteracted by the HIV-1 accessory gene product Nef. Here, we engineered, via CRISPR/Cas9 technology, T-cell lines that express endogenous *SERINC5* alleles tagged with a knocked-in HA epitope. This genetic modification enabled us to study basic properties of endogenous SERINC5 and to verify proposed mechanisms of HIV-1 Nef-mediated counteraction of SERINC5. Using this unique resource, we identified the susceptibility of endogenous SERINC5 protein to posttranslational modulation by type I IFNs and suggest uncoupling of Nef-mediated functional antagonism from SERINC5 exclusion from virions.

## INTRODUCTION

During its replication cycle, HIV-1 needs to overcome and counteract several intracellular restrictions exerted by cellular antiviral restriction factors. The HIV-1 accessory protein Nef, which is essential for the generation of fully infectious HIV-1 particles, especially in selected lymphoid T-cell lines (1) and primary T cells (2), antagonizes the restriction imposed by cellular SERINC5 (3, 4) and, to a lesser extent, SERINC3 (4) proteins. Murine leukemia virus (MLV) and equine infectious anemia virus (EIAV) have evolved cognate SERINC5-counteracting factors, glyco-Gag and S2, respectively (3, 5–8), illustrating the importance of developing antagonistic strategies against SERINC5 during virus evolution. The antiviral mode of action of SERINC5 and the mechanisms of Nef-mediated counteraction have been addressed in several studies using heterologous expression systems based on transient overexpression and genetic knockout systems (3, 4, 9–18). In these assays, SERINC5’s ability to reduce the infectivity of HIV-1 Δ*nef* particles is associated with its incorporation into virions, which reduces the Env-mediated capacity to fuse with target cells during the next round of infection. This has spurred the concept that SERINC5 interferes quantitatively and/or qualitatively with the HIV-1 Env-mediated membrane fusion process through inactivation of HIV-1 Env glycoproteins, resulting in diminished fusion pore formation (11, 12). HIV-1 Nef downregulates cell surface levels of ectopically expressed SERINC5 (3, 4, 10) and may target it for lysosomal degradation (17), thus diminishing quantities of surface SERINC5 protein pools. However, some data also point toward additional modes of counteraction, including inactivation of residual virus-associated SERINC5 molecules (10). The regulation of the expression and subcellular localization of SERINC5 protein remains largely unexplored to date. The impact that Nef exerts on physiological levels of endogenously expressed SERINC5 protein and how these relate to functional counteraction of antiviral restriction are unclear. Here, by applying a CRISPR/Cas9-assisted epitope knock-in strategy, we investigate the fate of endogenous SERINC5 protein in uninfected and HIV-1-infected Jurkat T cells, explore aspects of regulation of SERINC5 protein expression, and probe existing concepts of SERINC5-imposed restriction and Nef-mediated antagonism of SERINC5.

## RESULTS

### CRISPR/Cas9-assisted generation of Jurkat T cells expressing HA epitope-tagged *SERINC5* alleles.

**FIG 1:**
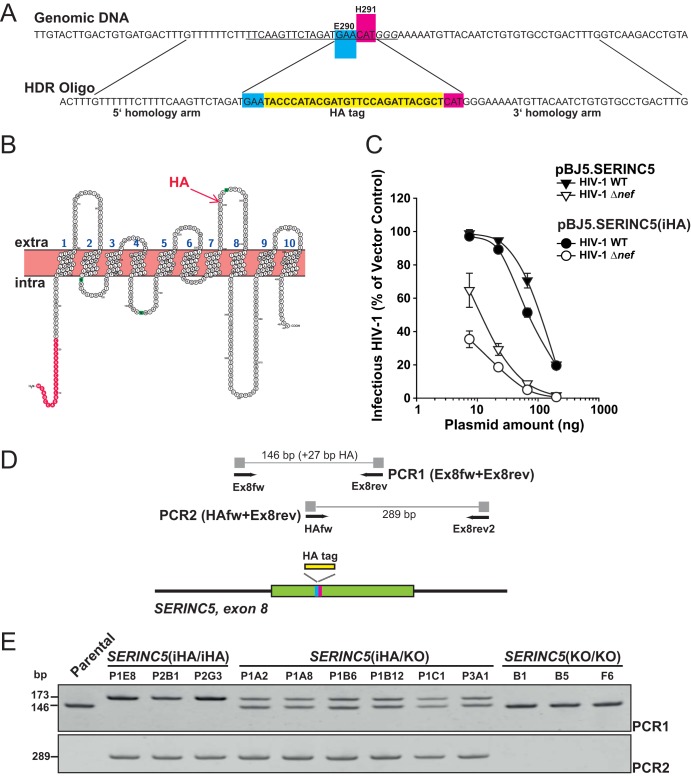
CRISPR/Cas9-assisted generation of Jurkat T cells expressing HA epitope-tagged *SERINC5* alleles. (A) Close-up of the CRISPR/Cas9-assisted homology-directed knock-in strategy. The guide RNA target sequence is underlined, with protospacer adjacent motif (PAM) in italics. HDR, homology-directed repair. (B) Putative topology of SERINC5 protein and indication of the HA epitope insertion (Protter) (31). (C) HA insertion at the interface of E^290^ and H^291^ of SERINC5 preserves the antiviral function of SERINC5 and its susceptibility to Nef-mediated counteraction. HEK293T cells were seeded into 12 wells and cotransfected with the indicated proviral DNAs and decreasing amounts of the indicated SERINC5-encoding plasmids (200 to 8 ng). At 2 days posttransfection, the infectivity of secreted virions was analyzed in a TZM-bl-based assay. Shown are the relative levels of infectivity, with 100% representing the infectivity of particles generated in vector-transfected controls. Values are arithmetic means ± SD from one representative experiment out of two independent experiments. (D) Illustration of the *SERINC5* genomic locus with primer binding sites and location of the HA tag. (E) Genomic DNA from parental Jurkat cells and Jurkat *SERINC5*(iHA/iHA), *SERINC5*(iHA/KO), and *SERINC5*(KO/KO) clones was analyzed by PCR as indicated (also illustrated in panel D), and products were separated on a 7.5% acrylamide–TBE gel. Shown are data from one representative experiment out of two independent experiments.

We set out to epitope tag endogenous *SERINC5* gene alleles in Jurkat T cells, which endogenously express *SERINC5* mRNA and represent an established model system for investigation of the long-appreciated Nef-mediated HIV-1 infectivity rescue (3, 4). By applying a CRISPR/Cas9-assisted strategy, we inserted the hemagglutinin (HA)-encoding sequence within exon 8 of the *SERINC5* gene (Fig. 1A), which is expected to result in the expression of endogenous SERINC5 protein displaying an HA tag within its fourth extracellular loop at the interface of E^290^ and H^291^ (Fig. 1B). This type of insertion has already been conducted in the context of heterologous expression from a *SERINC5*-encoding plasmid (4), and we confirmed that HA tagging at this position preserves the antiviral activity and susceptibility to Nef-mediated counteraction of SERINC5 (Fig. 1C). DNA from individual clones was verified using a PCR strategy that allows distinguishing the insertion of the HA-encoding sequence in one and two *SERINC5* gene alleles, respectively (Fig. 1D and E). Both *SERINC5* alleles were partially genotyped by Sanger sequencing (Table 1). We obtained two types of Jurkat clones: *SERINC5*(iHA/iHA) clones expressed HA in both *SERINC5* alleles, whereas *SERINC5*(iHA/KO) clones expressed HA in only one allele, while the second allele bore a frameshift or deletion (Table 1).

**TABLE 1:** *SERINC5* genotypes of clones used in this study^*a*^

Genotype	Clone	Allele 1 insertion	Allele 1 sequence	Allele 2 insertion	Allele 2 sequence
KO/KO	B1	+10 bp	GCCCCAGGAGTTTTGCATCCCCTCA	+1 bp	GCCCCAGGACTTCGGCAGTCCC
KO/KO	B5	+5 bp	GCCCCAGGAGGGCCTTCGGCAGTCCCTCA	+4 bp	GCCCCAGGAGGACTTCGGCAGTCCCTCA
KO/KO	F6	+8 bp	GCCCCAGGAGGCCCCCTTTCGGCAGTCCC	+1 bp	GCCCCAGGAATTCGGCAGTCCC
iHA/KO	P1A2	iHA tag	GATGAATACCCATACGATGTTCCAGATTACGCTCATGGG (HA integrated)	+2 bp	TTCTAGATGAACCCATGGGAAA
iHA/KO	P1A8	iHA tag	GATGAATACCCATACGATGTTCCAGATTACGCTCATGGG (HA integrated)	+1 bp	CTAGATGAACTATGGGAA
iHA/KO	P1B6	iHA tag	GATGAATACCCATACGATGTTCCAGATTACGCTCATGGG (HA integrated)	+2 bp	CTAGATGACCACATGGG
iHA/KO	P1B12	iHA tag	GATGAATACCCATACGATGTTCCAGATTACGCTCATGGG (HA integrated)	+2 bp	CTAGATGAATCCATGGG
iHA/KO	P3A1	iHA tag	GATGAATACCCATACGATGTTCCAGATTACGCTCATGGG (HA integrated)	+1 bp	TAGATGAAACATGGGAAAA
iHA/KO	P1C1	iHA tag	GATGAATACCCATACGATGTTCCAGATTACGCTCATGGG (HA integrated)	+5 bp	TCTAGATGAAGGCCCTACCCA
HA/HA	P1E8	iHA tag	GATGAATACCCATACGATGTTCCAGATTACGCTCATGGG (HA integrated)	iHA tag	GATGAATACCCATACGATGTTCCAGATTACGCTCATGGG (HA integrated)
HA/HA	P2B1	iHA tag	GATGAATACCCATACGATGTTCCAGATTACGCTCATGGG (HA integrated)	iHA tag	GATGAATACCCATACGATGTTCCAGATTACGCTCATGGG (HA integrated)
HA/HA	P2G3	iHA tag	GATGAATACCCATACGATGTTCCAGATTACGCTCATGGG (HA integrated)	iHA tag	GATGAATACCCATACGATGTTCCAGATTACGCTCATGGG (HA integrated)

a Underlining indicates insertions.

### Expression of endogenous *SERINC5* mRNA and SERINC5 protein.

**FIG 2:**
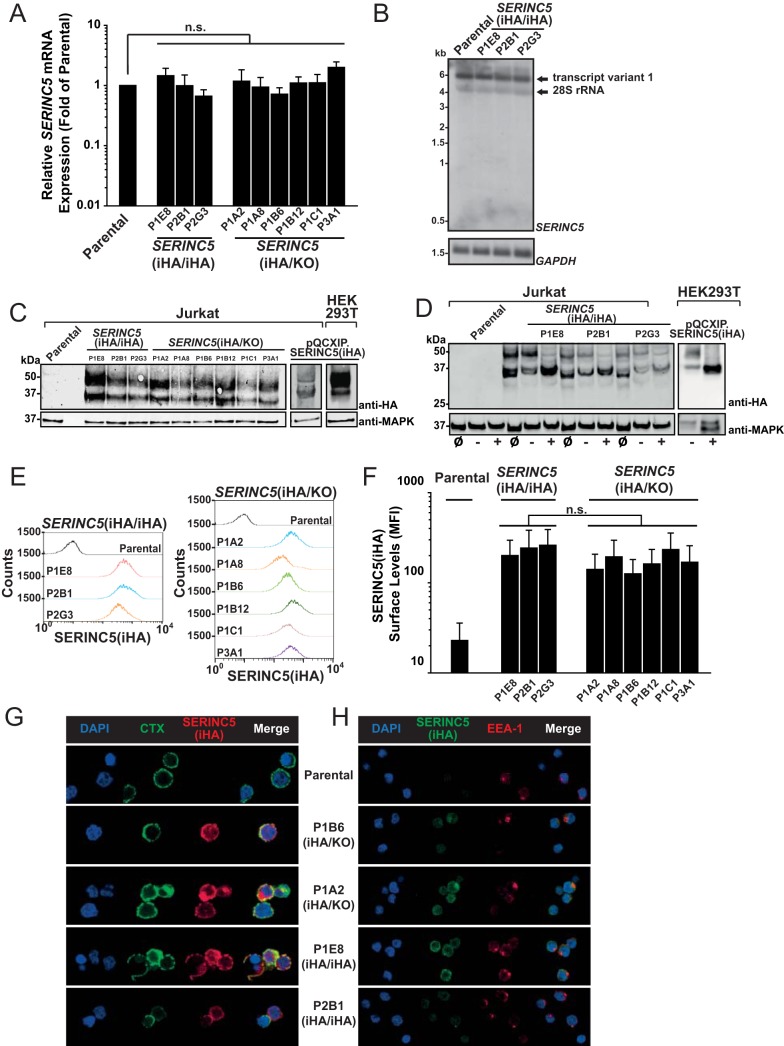
Characterization of expression of endogenous SERINC5 protein and *SERINC5* mRNA. (A) Q-RT-PCR analysis of *SERINC5* mRNA expression in parental Jurkat T cells and the indicated clones. *SERINC5* mRNA expression in parental T cells was set to a value of 1. Error bars indicate SEM of data from three independent experiments. (B) Total RNA from parental T cells and the indicated clones was subjected to Northern blotting using *SERINC5*- and *GAPDH*-specific probes. Shown are data from one representative experiment out of two independent experiments. (C) Lysates from the indicated cell lines and clones were subjected to anti-HA and anti-MAPK immunoblotting. Shown are data from one representative experiment out of three independent experiments. (D) Lysates from the indicated cell lines and clones were loaded either directly (input [ø]) or following mock digestion (−) or PNGase digestion (+) in glycoprotein-denaturing buffer. (E) The indicated cell lines and clones were immunostained for HA surface expression and analyzed by flow cytometry. Shown are representative histograms from one experiment out of three independent experiments. (F) Quantification of the mean fluorescence intensity (MFI) of SERINC5(iHA) surface expression. Error bars indicate SEM from three experiments, including the one shown in panel D. (G) The indicated clones were stained with CTX-FITC, followed by anti-CTX antibody and anti-HA antibody staining. Cells were then PFA fixed and analyzed by confocal microscopy. Shown are data from one representative experiment out of three independent experiments. (H) The indicated clones were PFA fixed, permeabilized, and immunostained with anti-HA and anti-EEA-1 antibodies. Shown are data from one representative experiment out of three independent experiments.

HA epitope tagging of endogenous *SERINC5* genes did not modulate *SERINC5* mRNA expression levels as assessed by quantitative real-time PCR (Q-RT-PCR) analysis using a primer-probe assay that amplifies a region common to all three known isoforms (19) (Fig. 2A). Furthermore, Northern blot experiments using a *SERINC5*-specific probe revealed the expression of isoform 1 in parental Jurkat T cells and in *SERINC5*(iHA/iHA) clones (Fig. 2B), suggesting that HA-encoding *SERINC5* mRNAs equal *SERINC5* mRNAs of parental Jurkat T cells in terms of abundance and splicing behavior. HA sequence knock-in enabled detection of endogenous SERINC5 protein by anti-HA immunoblotting of cell lysates (Fig. 2C). Parental Jurkat T cells were negative for HA, and lysates from *SERINC5*(iHA/iHA) and *SERINC5*(iHA/KO) clones displayed migration patterns that were identical to those of lysates obtained from Jurkat T cells and HEK293T cells transduced with SERINC5(iHA). SERINC5(iHA) presented as two protein species of 51 and 35 kDa, respectively. The 51-kDa, but not the 35-kDa, species was sensitive to peptide-*N*-glycosidase (PNGase) digestion (Fig. 2D), suggesting that it corresponds to the reported molecular weight of fully N-glycosylated protein (16) and that the 35-kDa species represents a nonglycosylated albeit still antiviral SERINC5 species (16). Endogenous SERINC5(iHA) was detectable at the surface of intact cells by flow cytometric analysis of anti-HA immunostaining (Fig. 2E and F), with a trend toward higher surface levels in *SERINC5*(iHA/iHA) clones than in most *SERINC5*(iHA/KO) clones. Immunofluorescence microscopy demonstrated presence of endogenous SERINC5(iHA) at the plasma membrane and colocalization with cholera toxin-stainable ganglioside GM1, suggesting an association with lipid rafts (Fig. 2G). Colocalization with the early endosomal marker EEA1 was only rarely observed (Fig. 2H).

### Type I interferon modulates cell surface expression of endogenous SERINC5 protein in the absence of modulation of mRNA and protein quantities.

**FIG 3:**
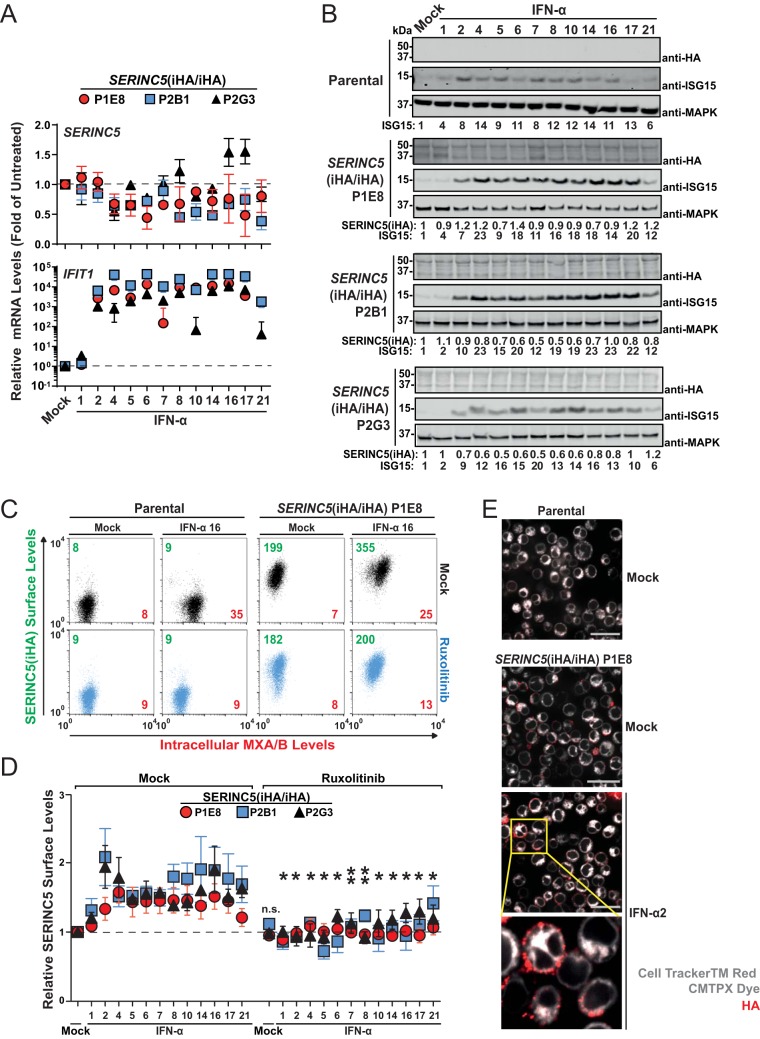
Type I interferon modulates cell surface expression of endogenous SERINC5 protein in the absence of modulation of mRNA and protein quantities. (A) Q-RT-PCR analysis of *SERINC5* and *IFIT1* mRNA expression in *SERINC5*(iHA/iHA) Jurkat clones 48 h after treatment with the indicated IFN-α subtypes (10 ng/ml) or mock treatment. *SERINC5* and *IFIT1* mRNA expression levels in mock-treated cells were set to a value of 1. Error bars indicate SEM of data from three independent experiments. (B) Parental cells and the indicated *SERINC5*(iHA/iHA) clones were treated with the indicated IFN-α subtypes (10 ng/ml) or mock treated. Lysates were then subjected to anti-HA, anti-ISG15, and anti-MAPK immunoblotting. Numbers indicate fold changes in levels of the indicated proteins. For each cell line, one representative immunoblot from two to three independent experiments is shown. (C) Parental cells and the *SERINC5*(iHA/iHA) clone P1E8 were treated for 18 h with ruxolitinib (10 μM) or mock treated, followed by treatment with the indicated with IFN-α subtypes (10 ng/ml) or mock treatment for an additional 48 h. Cells were then immunostained with anti-HA for surface SERINC5(iHA), PFA fixed, permeabilized, and immunostained for intracellular MXA/B. Numbers indicate mean fluorescence intensities for SERINC5(iHA) (green) and for MXA/B (red). Shown are representative dot plots from one experiment out of six independent experiments. (D) Quantification of relative cell surface SERINC5(iHA) protein expression in the indicated *SERINC5*(iHA/iHA) clones. Error bars indicate SEM of data from two to six independent experiments, including the one shown in panel C. Statistical analysis refers to each individual interferon subtype in the absence and presence of ruxolitinib. (E) The *SERINC5*(iHA/iHA) clone P1E8 was treated for 48 h with IFN-α2a (250 U/ml), labeled with CellTracker red CMTPX dye, and immunostained with anti-HA. Cells were then PFA fixed and analyzed by confocal microscopy. Mock-treated parental cells are shown as a specificity control.

In line with previously reported work (3, 4), treatment of *SERINC5*(iHA/iHA) clones with individual interferon alpha (IFN-α) subtypes failed to consistently upregulate *SERINC5* mRNA expression, while *IFIT1* mRNA expression was induced by 40- to 44,000-fold by all active IFN-α subtypes, confirming the effectiveness of the IFN-α treatment (Fig. 3A). Of note, individual IFN-α subtypes have been reported to substantially vary in their antiviral potencies (20), providing a rationale for testing them individually and side by side. IFN-α1, which has a comparably low affinity for IFN-alpha/beta receptor (IFNAR) (21), was largely inactive under these experimental conditions. Additionally, no consistent changes in total levels of cell-associated SERINC5(iHA) protein were detected upon IFN-α treatment, arguing against a potential impact of IFN-α on SERINC5 protein synthesis and overall stability. ISG15 protein levels were upregulated 4- to 23-fold by individual active IFN-α subtypes (Fig. 3B). Interestingly, amounts of SERINC5 at the surface of *SERINC5*(iHA/iHA) T cells increased up to 2-fold upon IFN-α treatment (Fig. 3C and D). IFN-α-induced enhancement of SERINC5(iHA) surface levels was entirely abrogated in the context of cotreatment with the Jak/STAT inhibitor ruxolitinib. As a reference, within identical samples, the induction of intracellular MXA/B expression, which is strictly IFN dependent (22), was up to 3.6-fold (Fig. 3C). Immunofluorescence microscopy of SERINC5(iHA) revealed HA positivity at the surface of both mock-treated and IFN-α-treated cells (Fig. 3E). Together, these data suggest that the subcellular localization of SERINC5 is regulated by type I IFNs in the absence of modulation of mRNA and protein quantities.

### HIV-1 Nef-mediated enhancement of particle infectivity may occur in the absence of exclusion of endogenous SERINC5 from virions.

**FIG 4:**
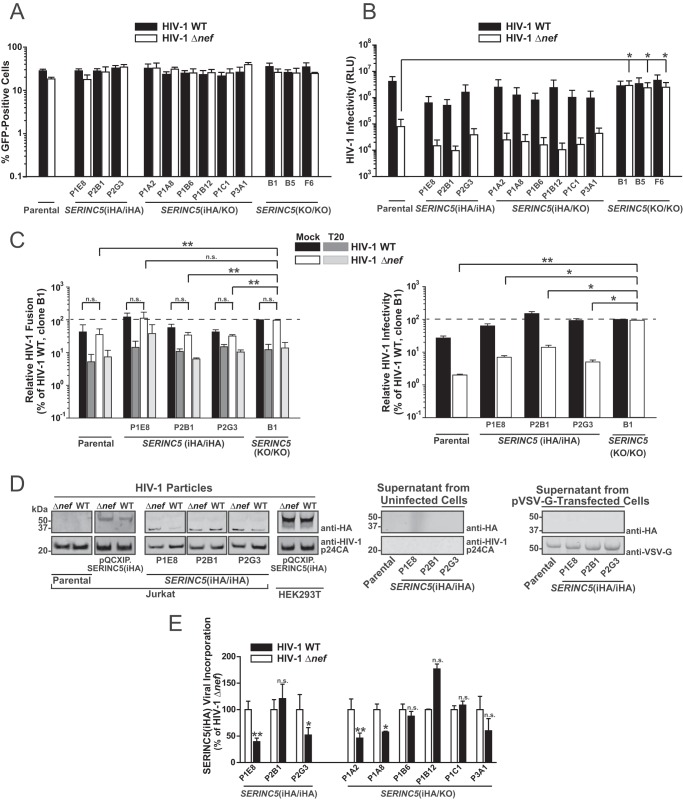
HIV-1 Nef-mediated enhancement of particle infectivity may occur in the absence of exclusion of endogenous SERINC5 from virions. (A) Parental Jurkat T cells and the indicated clones were infected with VSV-G-pseudotyped HIV-1 WT IRES-GFP and HIV-1 Δ*nef* IRES-GFP. At 2 days postinfection, cells were analyzed for GFP expression by flow cytometry. Error bars indicate SEM from three independent experiments. (B) Infectivity of virions secreted from infected cells (shown in panel A) was analyzed in a luminometric TZM-bl infectivity assay. Shown are SEM from three independent experiments (corresponding to the data in panel A). RLU, relative luminescence units. (C) Parental cells, the indicated *SERINC5*(HA/HA) clones, and *SERINC5*(KO/KO) clone B1 were electroporated with a plasmid encoding the β-lactamase-Vpr chimeric fusion protein (pBlaM-Vpr) and proviral plasmids encoding WT HIV-1 or the HIV-1 Δ*nef* mutant. Concentrated virus-containing supernatants were added, in the presence or absence of the HIV-1 fusion inhibitor T-20, to TZM-bl cells, and fusion was quantified as the change in the fluorescence emission of the cell-permeable CCF2 substrate upon cleavage by BlaM-Vpr by flow cytometry. (Left) Relative HIV-1 fusion, normalized to that of WT HIV-1, of clone B1; (right) corresponding relative HIV-1 infectivity. Shown are SEM from two to three independent experiments. (D) Concentrated supernatants from infected, uninfected, and pVSV-G-transfected cells were subjected to immunoblotting with the indicated antibodies. (E) Relative levels of HIV-1 p24-associated SERINC5(iHA) protein were quantified by Odyssey infrared-based imaging. Shown are SEM from three independent experiments.

Key concepts of SERINC5’s antiviral mode of action and of its counteraction by HIV-1 Nef were established mainly in heterologous expression systems. Here, we aimed at testing these working models in the context of endogenously expressed SERINC5(iHA) protein. Parental Jurkat T cells and the *SERINC5*(iHA/iHA), *SERINC5*(iHA/KO), and *SERINC5*(KO/KO) clones shared similar susceptibilities to infection by the vesicular stomatitis virus G protein (VSV-G)-pseudotyped, internal ribosome entry site (IRES)-green fluorescent protein (GFP)-expressing wild-type (WT) HIV-1 and HIV-1 Δ*nef* mutant, as assessed by analysis of the percentage of GFP-positive cells at 2 days postinfection (Fig. 4A). Infected parental cells and the *SERINC5*(iHA/iHA) and *SERINC5* (iHA/KO) clones produced wild-type HIV-1 particles of similar infectivities and shared the requirement of proviral Nef expression for the generation of fully infectious particles (Fig. 4B). As expected, CRISPR/Cas9-mediated knockout of both *SERINC5* alleles fully rescued the ability of Jurkat T cells to generate infectious HIV-1 Δ*nef* virions (Fig. 4B). Interestingly, the reduction of HIV-1 Env-dependent, T-20-sensitive fusogenicity (Fig. 4C, left) that accompanied the SERINC5-mediated reduction of particle infectivity (Fig. 4C, right) was relatively modest, if even detectable, indicating that additional, postfusion restrictions might be exerted by SERINC5. In addition, the fusion capabilities of HIV-1 wild-type and HIV-1 Δ*nef* virions were very similar, suggesting that functional counteraction of SERINC5 by Nef does not occur at the level of virus-cell fusion. Immunoblotting of sucrose cushion-purified virus followed by quantitative infrared-based imaging revealed an association of the 35-kDa, but not of the 51-kDa, protein species of endogenous SERINC5(iHA) protein in HIV-1 Δ*nef* particles derived from all Jurkat HA knock-in clones (Fig. 4D). In contrast, virions derived from Jurkat T cells and HEK293T cells that had been retrovirally transduced with SERINC5(iHA) presented exclusively and predominantly the 51-kDa species, respectively (Fig. 4D), in accordance with previously reported results (4, 16). Surprisingly, proviral Nef expression appeared to not reduce SERINC5 virion incorporation in all HA knock-in clones (Fig. 4D and E). Virion-associated levels of SERINC5(iHA) were significantly reduced by 50 to 60% in wild-type HIV-1 particles (compared to HIV-1 Δ*nef* particles) derived from the *SERINC5*(iHA/iHA) clones P1E8 and P2G3, the *SERINC5*(iHA/KO) clones P1A2 and P1A8, and Jurkat T cells transduced with SERINC5(iHA). In contrast, levels of SERINC5(iHA) were unaffected or even enhanced by Nef in virions derived from the SERINC5(iHA/iHA) clone P2B1 and the SERINC5(iHA/KO) clones P1B6, P1B12, P1C1, and P3A1 (Fig. 4D and E). The fact that proviral HIV-1 Nef neither modulated SERINC5(iHA) incorporation into virions generated by SERINC5(iHA)-transduced HEK293T cells nor counteracted the SERINC5-imposed restriction at the infectivity level (WT average, 100% [standard error of the mean {SEM}, 26.8%]; Δ*nef* average, 95.6% [SEM, 20.5%] [not significant]) is probably due to the saturating expression levels of SERINC5. Importantly, supernatants from uninfected or pVSV-G-transfected SERINC5(iHA/iHA) clones did not display HA positivity (Fig. 4D), arguing against an accumulation of SERINC5(iHA) in extracellular vesicles that may have cosedimented in our virus particle preparations (23). These results suggest that endogenous SERINC5 protein, at least the low-molecular-weight form, which has been suggested to exert antiviral activity (16), is incorporated into HIV-1 Δ*nef* particles at detectable levels. However, Nef expression in virus-producing cells may not necessarily decrease endogenous SERINC5 protein association into virions despite exerting a strong antagonistic activity at the functional level.

### HIV-1 Nef modulates subcellular localization and trafficking of endogenous SERINC5 protein.

**FIG 5:**
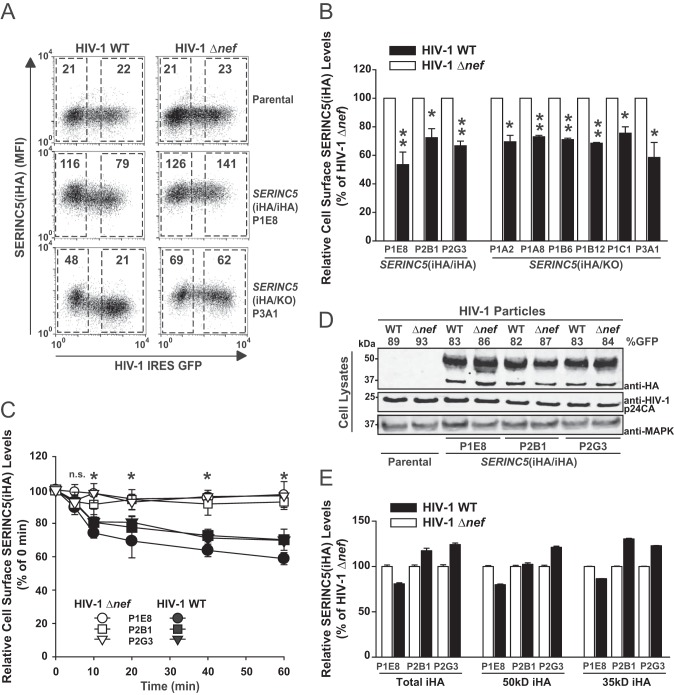
HIV-1 Nef modulates the subcellular localization and trafficking of endogenous SERINC5. (A) Parental Jurkat T cells and the indicated clones were infected with the indicated VSV-G-pseudotyped viruses. At 2 days postinfection, intact cells were immunostained for surface HA prior to PFA fixation and analyzed for SERINC5(iHA) surface expression by flow cytometry. In the dot plots, the SERINC5(iHA) surface levels are plotted against GFP. Numbers inside the gates indicate the MFI of the surface SERINC5(iHA) signal. Shown are representative dot plots from one experiment out of three independent experiments. (B) Relative level of SERINC5(iHA) surface expression. The MFI for cell surface-expressed SERINC5(iHA) was quantified on GFP-positive cells in the R3 gate relative to the MFI of GFP-negative cells in the R2 gate. Values obtained for cells infected with the HIV-1 Δ*nef* mutant were set to 100%. Error bars indicate SEM from three experiments. (C) HIV-1 Nef enhances the rate of endocytosis of endogenous SERINC5(iHA). Shown are kinetics of the decrease of surface-exposed SERINC5 in *SERINC5*(iHA/iHA) clones infected with WT HIV-1 or the HIV-1 Δ*nef* mutant. The values at time zero were set to 100%. Error bars indicate SEM from three experiments. (D) Parental cells and the indicated clones were infected with the indicated VSV-G-pseudotyped HIV-1 at a high MOI. The percentage of GFP-positive cells is indicated. At 2 days postinfection, cell lysates were subjected to immunoblotting with anti-HA, anti-HIV-1 p24CA, and anti-MAPK antibodies. Shown is one representative blot from one experiment out of three independent experiments. (E) Relative levels of cell-associated SERINC5 protein in infected cells were quantified by Odyssey infrared-based imaging. Shown is the quantification of the total HA signal, of the 51-kDa band only, and of the 35-kDa band only. Error bars indicate SEM from three independent experiments.

We next dissected the impact of HIV-1 Nef on endogenous SERINC5 protein surface localization in virus-producing cells. Flow cytometric analysis of infected *SERINC5*(iHA/iHA) and *SERINC5*(iHA/KO) clones demonstrated the susceptibility of the endogenous protein to downregulation by HIV-1 Nef (Fig. 5A and B). The magnitude of reduction from the plasma membrane ranged between 25 and 47% in individual clones (Fig. 5B). Furthermore, HIV-1 Nef increased the rate of internalization of endogenous surface SERINC5 in a kinetic endocytosis assay in all *SERINC5*(iHA/iHA) clones (Fig. 5C). Infection of cells at a high multiplicity of infection (MOI), however, failed to result in detectable quantities in both SERINC5(iHA) protein species (Fig. 5D and E), arguing against Nef-induced degradation of the antiviral factor. These results establish that proviral HIV-1 Nef modulates the cell surface expression and the rate of internalization of endogenous SERINC5 protein in the absence of an alteration of steady-state protein levels.

## DISCUSSION

Functional studies in genetically modified T cells have shed light on the importance of *SERINC5* in HIV-1 restriction and the ability of Nef to antagonize this antiviral factor (3, 4). However, due to the current unavailability of an anti-SERINC5 antibody of sufficient sensitivity and specificity, we lack a good understanding of endogenous SERINC5 protein expression and subcellular localization. Concepts of its antiviral mode of action and of its counteraction by viral antagonists were derived, to a large extent, from experiments in which SERINC5 was ectopically expressed. However, knowledge gained through the heterologous expression of cellular factors does not necessarily reflect key aspects of the endogenous protein. This can be attributed to the absence of a natural gene expression context and/or to nonphysiological expression levels. Previous studies reported aberrant effects resulting from SERINC5 overexpression and stressed the importance of expressing SERINC5 at low levels using plasmids from which SERINC5 expression is driven by a low-activity promoter (4) in order to attain physiological expression levels.

Our work establishes predominant cell surface expression of endogenous SERINC5 with a high degree of association with lipid rafts. Steady-state intracellular SERINC5 expression was detectable but did not display consistent colocalization with the early endosomal marker EEA1, suggesting that the natural recycling pathway of SERINC5 does not involve early endosomes. Functional inactivation of one *SERINC5* allele, resulting in SERINC5 expression exclusively from the second, remaining allele, did not significantly alter SERINC5 expression levels and, thus, did not modulate the antiviral capacity of Jurkat T cells, suggesting that the loss of one functional allele is compensated for by the other, functional, allele. Interestingly, type I IFN increased the abundance of surface SERINC5 in an entirely Jak/STAT inhibitor-sensitive manner without augmenting mRNA and whole-cell-associated protein quantities. This suggests that type I IFN treatment induces a relocalization of intracellular SERINC5 to the plasma membrane and/or stabilizes cell surface SERINC5 by impairing or retarding its endocytosis or recycling. Although the magnitude of enhancement of surface SERINC5 levels by type I IFN was lower than that of the induction of MXA/B protein expression, it may suffice to limit the efficiency of Nef-mediated antagonism. However, due to the multitude of IFN-stimulated antiviral genes that are induced by IFN treatment and that decrease the efficiency of several steps of the HIV-1 replication cycle, including tetherin-mediated retention of virus particles (24, 25) and 90K-mediated reduction of particle infectivity (26), experiments aiming at testing of this hypothesis have been inconclusive so far. To our knowledge, this is the first example of an antiviral factor whose subcellular localization is modulated at the posttranslational level by type I IFNs, and future studies deciphering the IFN-induced interactome of endogenous SERINC5 are required to delineate the underlying mechanism.

Previous work suggested that SERINC5’s antiviral activity is largely driven by its association with virions, thereby modifying them in a manner that results in inefficient Env-mediated membrane fusion with target cells. However, the SERINC5-imposed reduction of Env-mediated fusion of BlaM-Vpr-containing viruses to target cells was relatively low (up to 3-fold) compared to the reduction of HIV-1 infectivity (up to 50-fold in the parallel infectivity assay). This observation has already been made in heterologous assays in which SERINC5-encoding plasmids were expressed at doses anticipated to approach physiological expression levels (3, 4) and is consistent with the possibility of additional, postfusion restrictions exerted by SERINC5.

Exclusion of SERINC5 from virions is supposed to be a consequence of its Nef-mediated downregulation from the cell surface (3, 4) and potentially its degradation in intracellular compartments (17). Indeed, endogenous SERINC3 has been shown to be excluded from virion incorporation in a Nef-dependent manner in a mass spectrometry-based approach (3). Here, we demonstrate that SERINC5 is susceptible to Nef-mediated downregulation from the cell surface and internalization in all tested clones. Nef-mediated reduction of surface levels of endogenous SERINC5 in infected T cells was statistically significant but <2-fold, which again appears to be relatively mild when set in relation to the much more pronounced, up to >250-fold enhancement of infectivity of released viruses. While targeting to lysosomal degradation by Nef has been suggested by others using heterologous expression systems (17), steady-state levels of endogenous SERINC5 remained unaltered in infected cells, irrespective of the Nef expression status. Furthermore, we demonstrate HA positivity associated with HIV-1 Δ*nef* virions, suggesting viral incorporation of SERINC5, as expected. HA positivity in virions was associated with a band of 35 kDa, and we failed to detect the high-molecular-weight SERINC5 species in virus preparations. In contrast, the exclusive and predominant incorporation of the 51-kDa species in virions generated by Jurkat T cells and HEK293T cells expressing transduced SERINC5(iHA) demonstrates that our assay displays sufficient sensitivity to detect this SERINC5 species in general and reproduces previously reported findings (4, 16). Along this line, SERINC5 clearly presented as two distinct species in cell lysates in our assay. Therefore, it is conceivable that the virus-associated HA signal derived from Jurkat *SERINC5*(iHA knock-in) clones represents impartially glycosylated SERINC5 species (16). In the context of heterologous expression, it has been suggested that a glycosylated species of SERINC5 with a molecular weight of 55 kDa is specifically incorporated into virions, whereas a low-molecular-weight form of SERINC5 of ≤40 kDa predominates in cell lysates and corresponds to a nonglycosylated protein (16). The same study showed that N-glycosylation of SERINC5 at residue N294 stabilized its steady-state levels and prevented otherwise rapid targeting for proteasomal degradation. Importantly, the nonglycosylated, low-molecular-weight form of SERINC5 maintained its antiviral capacity and its susceptibility to Nef-mediated counteraction. Whether SERINC5, when expressed from its endogenous promoter and under physiological conditions, is susceptible to N-linked glycosylation with efficiencies and kinetics similar to those of heterologously expressed SERINC5 remains an interesting question whose answer might help to reconcile the apparently divergent results obtained in heterologous and endogenous expression systems in the future.

While proviral Nef expression was uniformly required for particle infectivity rescue in all SERINC5-expressing clones, it reduced virus-associated HA positivity in particles in only four out of nine clones. Clone-specific properties at the genetic or transcriptional level that specifically modulate the susceptibility of SERINC5 to Nef-mediated exclusion from virions may cause this heterogeneity. Indeed, transcriptome sequencing (RNA-seq) analysis revealed a set of genes that are differentially expressed in the two phenotypic groups of clones. Regardless of the underlying reason, the apparent dispensability of Nef-mediated exclusion of endogenous SERINC5 from virions for infectivity rescue provides evidence for the presence of additional counteractive mechanisms of Nef, directed against virus-associated pools of SERINC5, as has been postulated by another group (10).

Together, data from this study establish CRISPR/Cas9-assisted epitope tagging of endogenous alleles of *SERINC5* as a useful technology that enabled us to investigate key aspects of SERINC5 antiviral restriction and HIV-1 Nef-mediated antagonism. A similar approach in other cell types in the future might reveal the extent to which our findings can be extrapolated in more physiologically relevant, primary T cells and macrophages. Future studies using this resource may help to advance our understanding of both SERINC5 restriction and viral counteraction and its physiological function.

## MATERIALS AND METHODS

### Cell lines.

HEK293T cells and Jurkat T cells were purchased from the ATCC and cultured as recommended. TZM-bl cells were obtained from the NIH AIDS Reagent Program. For the generation of *SERINC5*(KO/KO) cell lines, Jurkat T cells were electroporated with 100 μg/ml of a plasmid expressing Cas9-2A-enhanced GFP (EGFP) and U6-driven chimeric guide RNA (*SERINC5* target sequence GCTGAGGGACTGCCGAATCC[TGG]) using the Neon transfection system (Thermo Fisher Scientific, Darmstadt, Germany) at 1,500 V for 30 ms, with 1 pulse. EGFP-positive cells were sorted on a BD FACSAria cell sorter and clonally expanded. Individual clones were genotyped by Sanger sequencing (SeqLab, Göttingen, Germany) of the PCR-amplified genomic locus (forward primer TGCTGTGTTGACCAGGCTAA and reverse primer GGCATTGGATCCTGGAAAGC). Individual alleles were deduced using the Poly Peak Parser tool (27) and, where applicable, allocated based on peak strength. *SERINC5*(iHA/iHA) and *SERINC5*(iHA/KO) Jurkat clones were generated by coelectroporation of 1.8 μM Cas9 RNPs (Alt-R Cas9 protein, *trans*-activating crRNA (tracrRNA), and CRISPR-RNA (crRNA) [target sequence, TTCAAGTTCTAGATGAACAT{*GGG*}]; IDT, Leuven, Belgium) and 5 μM single-stranded DNA (ssDNA) repair oligonucleotide (ACTTTGTTTTTTCTTTTCAAGTTCTAGATGAATACCCATACGATGTTCCAGATTACGCTCATGGGAAAAATGTTACAATCTGTGTGCCTG [the HA tag is underlined]) using the Neon transfection system (1,500 V for 10 ms, with 3 pulses). Single-cell clones were screened by PCR (for PCR 1, for the presence of the HA tag, HA forward primer TACCCATACGATGTTCCAGATTA and HA reverse primer AGTTCACGCTCTTCGCCTTT; for PCR 2, for insert size, forward primer CTTCTGTGCGTTACAACTGGCC and reverse primer TAGTCACCAAGTTTTCATCTCTGTACAGG), followed by Tris-borate-EDTA (TBE)-PAGE (7.5%), and genotyped by Sanger sequencing of the PCR-amplified genomic locus (forward primer TGGCACTGAGCTGGAATCTG and reverse primer AGTTCACGCTCTTCGCCTTT).

### Plasmids, lentiviral vectors, and virus.

The retroviral vector pQCXIP.SERINC5(iHA) was generated by subcloning SERINC5(iHA) from pBJ5-SERINC5(iHA) (kindly provided by Heinrich Göttlinger) (4) into pQCXIP using NotI and AgeI restriction sites. Transduced cells were maintained as bulk cultures and expressed SERINC5 stably. The proviral DNAs pNL4.3 WT IRES-GFP and pNL4.3 Δ*nef* IRES-GFP (28) were kindly provided by Frank Kirchhoff. pVSV-G was kindly obtained by Oliver Keppler.

### Reagents.

IFN-α2a (Roferon) (used in Fig. 3E) was purchased from Roche. IFN-α subtypes were produced and purified as previously mentioned (20). Fluorescein isothiocyanate (FITC)-conjugated cholera toxin (CTX) was purchased from Sigma-Aldrich. T-20 was obtained from the NIH AIDS Reagent Program. Ruxolitinib was purchased from InvivoGen. CellTracker red CMTPX dye was purchased from Thermo Fisher Scientific.

### Quantitative RT-PCR.

Total RNA extraction from cells and DNase treatment were performed with a Maxwell LEV simplyRNA purification kit (Promega), followed by cDNA synthesis (NEB, Invitrogen). Quantification of relative *SERINC5* and *IFIT1* mRNA levels was performed with the 7500 Fast real-time PCR system (Applied Biosystems) using TaqMan PCR technology with premade primer-probe kits (Applied Biosystems). Relative mRNA levels were determined using the ΔΔ*C_T_* method, with human *RNASEP* mRNA (Applied Biosystems) as an internal reference. Each sample was analyzed in triplicates. Data analysis was performed using Applied Biosystems 7500 Fast system software.

### Northern blotting.

RNA extraction, gel electrophoresis, blotting, and detection with a radiolabeled probe were performed as described previously (29) but with the following adjustments. Ten micrograms of total RNA for each cell line was loaded onto the gel. The probes were labeled with the DecaLabel DNA labeling kit (Thermo Fisher Scientific), and the membrane was exposed to Amersham Hyperfilm MP (GE Healthcare) for 8 days (in the case of the SERINC5-specific probe) or for 8 h (glyceraldehyde-3-phosphate dehydrogenase [GAPDH]-specific probe) at −80°C. The SERINC5-specific probe of 870 bp was prepared by digestion of a plasmid bearing the *SERINC5* cDNA with XbaI and NotI. For the loading control, a GAPDH plasmid (a gift from K. Habers, Heinrich-Pette-Institut, Hamburg, Germany) was digested by EcoRI, generating a 1.3-kbp GAPDH-specific probe.

### Immunoblotting.

Cells were lysed with radioimmunoprecipitation assay (RIPA) buffer containing a protease inhibitor cocktail (Sigma). Virus particles were concentrated by ultracentrifugation through a 20% sucrose cushion and lysed using 1% Triton. Laemmli sample buffer was added to cell and virus lysates before loading. Proteins were run on a 10% SDS-PAGE gel and transferred onto nitrocellulose using a semidry transfer system (Biometra). Blocked membranes were incubated with the following primary antibodies: rabbit anti-mitogen-activated protein kinase (MAPK) (Santa Cruz), mouse anti-HA (clone HA-7; Sigma), mouse anti-p24 (ExBio), rabbit anti-HIV-1 p24CA (ExBio), mouse anti-interferon-stimulated gene 15 (ISG15) (clone F-9; Santa Cruz Technologies), and mouse anti-VSV-G (Santa Cruz) (a kind gift from Hanna-Mari Baldauf). Secondary antibodies conjugated to Alexa Fluor 680/800 fluorescent dyes were used for detection and quantification with the Odyssey infrared imaging system (Li-Cor Biosciences).

### PNGase treatment.

Cells were lysed with RIPA buffer containing a protease inhibitor cocktail (Sigma). Cell lysates were either left untreated (input) or pretreated with 1× Glycobuffer 2 (New England Biolabs) and 1% NP-40 buffer (New England Biolabs), in the presence or absence of 500 U of PNGase F (New England Biolabs). The mixture was incubated for 1 h at 37°C. Laemmli sample buffer was added to lysates before loading for immunoblotting.

### Immunofluorescence microscopy.

In order to visualize rafts together with SERINC5(iHA) (Fig. 2G), parental cells or *SERINC5*(iHA) clones were incubated with 25 μg/ml of FITC-conjugated CTX in 0.1% fetal bovine serum (FBS)–phosphate-buffered saline (PBS) buffer for 30 min on ice. Cross-linking was then performed by incubation with anti-CTX antibody at a 1:200 dilution for 30 min at 4°C and for 10 min at 37°C. Cells were then stained with anti-HA antibody (clone 3F10; Roche) at a 1:100 dilution, followed by an anti-rat Alexa Fluor 647-conjugated antibody, and fixed with 4% paraformaldehyde (PFA) for 5 min. To visualize the SERINC5(iHA) intracellular localization and the early endosomal compartment (Fig. 2H), cells were first fixed with 4% PFA for 5 min. Anti-HA and anti-EEA1 (clone 1G11; EBioscience) antibodies were diluted 1:300 and 1:100, respectively, in 0.1% FBS–0.05% saponin–PBS buffer, and cells were stained for 30 min at 4°C. Cells were then washed, specific secondary antibodies were diluted in the same staining buffer, and cells were colored for 30 min at 4°C. At the end of the staining procedures, cells were seeded onto poly-l-lysine-coated coverslips and mounted using a mounting medium containing 4′,6-diamidino-2-phenylindole (DAPI). For analysis, images were obtained with a confocal laser scanning microscope (LSM 700 upright; Leica). All images were processed by using the Fiji-ImageJ Magic Montage plug-in. In order to visualize SERINC5 surface expression upon IFN-α treatment, cells were incubated with CellTracker red CMTPX dye (1 μM) in Dulbecco’s modified Eagle’s medium (DMEM) for 15 min at 37°C. Cells were then washed and immunostained with anti-HA (clone 16B12; BioLegend) in PBS for 20 min at 4°C. After washing, cells were stained with the secondary antibody Alexa Fluor 647-conjugated goat anti-mouse IgG (Invitrogen) in PBS for 20 min at 4°C. After PFA fixation, cells were spinoculated in poly-l-lysine-coated 8-well chamber slides (Ibidi) for 5 min at 500 × *g*. Agarose (0.8%) in Fluorobrite DMEM was added carefully. For analysis, images were obtained with a confocal spinning-disk Ti-E microscope (Nikon/Andor). All images were processed by using the Fiji-ImageJ Magic Montage plug-in.

### SERINC5(iHA) surface staining and intracellular MXA/B staining.

Cells were immunostained with the primary mouse anti-HA antibody (clone 16B12; BioLegend), followed by secondary staining with Alexa Fluor 647-conjugated goat anti-mouse IgG (Invitrogen). After surface HA staining, cells were PFA fixed and analyzed for surface HA and GFP by flow cytometry. In selected experiments, surface HA staining was followed by Triton-mediated permeabilization and intracellular MXA/B staining using rabbit anti-MXA/B (Santa Cruz Biotechnology) as a primary antibody and Alexa Fluor 488-conjugated goat anti-rabbit IgG (Invitrogen). Flow cytometry analysis was performed using a FACSCalibur system with BD CellQuest Pro 4.0.2 software (BD Pharmingen) and FlowJo V10 software (FlowJo).

### TZM-bl infectivity assay.

The virus-containing supernatant was transferred to TZM-bl cells stably expressing long terminal repeat (LTR)-driven luciferase. Forty-eight hours later, cells were washed once with PBS and lysed using cell culture lysis buffer (Promega), and the Tat-dependent increase of luciferase enzyme activity in cell lysates was determined with a luciferase assay system (Promega). Luminometric activity was analyzed with a Centro LB 960 microplate luminometer and Ascent software 2.0.

### BlaM-Vpr fusion assay.

Jurkat T cells were electroporated (250 V at 1,000 μF) using GenePulser Xcell (Bio-Rad) with 9 μg pNL4.3 WT or pNL4.3 *Δnef* proviral DNA, 3 μg BlaM-Vpr DNA, and 1 μg pAdvantage (30). The virus-containing supernatant was collected at 48 h postelectroporation, ultracentrifuged through a sucrose cushion, and used for spinoculation of TZM-bl cells (1 h at 32°C). Following an additional 4 h at 37°C, cells were washed with CO_2_-independent medium (Sigma-Aldrich) and loaded with CCF2-AM dye (LiveBLAzer FRET-B/G loading kit) prepared as indicated by the manufacturer. Briefly, each sample was loaded with a 100-μl solution containing 0.2 μl CCF2-AM dye (Thermo Fisher Scientific), 0.8 μl solution B (Thermo Fisher Scientific), 1 μl of probenecid (2.5 mM; Thermo Fisher Scientific), and 98 μl CO_2_-independent medium. Cells were incubated in the dark at room temperature overnight. The percentage of cells undergoing fusion with BlaM-Vpr-containing virus particles was monitored by analysis of the change from green (520 nm) to blue (450 nm) fluorescence, using the fluorescence-activated cell sorter (FACS) LSRIII system with DIVA software, and analyzed using FlowJo V10 software (FlowJo).

### Internalization assay.

Infected T cells were immunostained on ice with the primary anti-HA antibody (clone 16B12; BioLegend). Cells were then shifted to 37°C for the indicated durations, placed back on ice, and immunostained on ice with secondary Alexa Fluor 647-conjugated goat anti-mouse IgG (Invitrogen), followed by PFA fixation. Flow cytometry analysis was performed using the FACSCalibur or FACS Accuri system with BD CellQuest Pro 4.0.2 software (BD Pharmingen) and FlowJo V10 software (FlowJo).

### Data presentation and statistical analysis.

If not otherwise stated, bars and symbols show the arithmetic means from the indicated number of repetitions. Error bars indicate standard deviations (SD) from one representative experiment out of at least three or SEM from the indicated number of individual experiments. Significance values were calculated using the 2-tailed Student *t* test and are indicated in the figures (*, *P < *0.05; **, *P < *0.01; n.s., not significant).
